# The Neglected Genes of ALS: Cytoskeletal Dynamics Impact Synaptic Degeneration in ALS

**DOI:** 10.3389/fncel.2020.594975

**Published:** 2020-11-13

**Authors:** María José Castellanos-Montiel, Mathilde Chaineau, Thomas M. Durcan

**Affiliations:** Early Drug Discovery Unit (EDDU), Montreal Neurological Institute-Hospital, McGill University, Montreal, QC, Canada

**Keywords:** ALS, ALS2, DCTN1, intermediate filaments, KIF5A, PFN1, SPAST, TUBA4A

## Abstract

Amyotrophic lateral sclerosis (ALS) is a neurodegenerative disease that selectively affects motor neurons (MNs) of the cortex, brainstem, and spinal cord. Several genes have been linked to both familial (fALS) and sporadic (sALS) cases of ALS. Among all the ALS-related genes, a group of genes known to directly affect cytoskeletal dynamics (*ALS2*, *DCTN1*, *PFN1*, *KIF5A*, *NF-L*, *NF-H*, *PRPH*, *SPAST*, and *TUBA4A*) is of high importance for MN health and survival, considering that MNs are large polarized cells with axons that can reach up to 1 m in length. In particular, cytoskeletal dynamics facilitate the transport of organelles and molecules across the long axonal distances within the cell, playing a key role in synapse maintenance. The majority of ALS-related genes affecting cytoskeletal dynamics were identified within the past two decades, making it a new area to explore for ALS. The purpose of this review is to provide insights into ALS-associated cytoskeletal genes and outline how recent studies have pointed towards novel pathways that might be impacted in ALS. Further studies making use of extensive analysis models to look for true hits, the newest technologies such as CRIPSR/Cas9, human induced pluripotent stem cells (iPSCs) and axon sequencing, as well as the development of more transgenic animal models could potentially help to: differentiate the variants that truly act as a primary cause of the disease from the ones that act as risk factors or disease modifiers, identify potential interactions between two or more ALS-related genes in disease onset and progression and increase our understanding of the molecular mechanisms leading to cytoskeletal defects. Altogether, this information will give us a hint on the real contribution of the cytoskeletal ALS-related genes during this lethal disease.

## Introduction

Amyotrophic lateral sclerosis (ALS) is a neurodegenerative disease characterized by the progressive loss of motor neurons (MNs) within the brain cortex, brainstem, and spinal cord. As MNs degenerate, the synaptic connections with their target muscles are lost, leading to muscle spasticity, weakness, and atrophy. Currently, there is no cure for ALS and available treatments only help to relieve symptoms. Typically, ALS patients die of respiratory failure within 2–5 years after diagnosis due to diaphragm paralysis. About 90–95% of ALS cases are sporadic (sALS) while the remaining 5–10% are familial (fALS) (Brown and Al-Chalabi, 2017; Volk et al., 2018).The clinical presentation of ALS is heterogeneous and sometimes it can be misdiagnosed with other MN diseases. For instance, the population of MNs involved and the survival can vary depending on the mutated gene and the specific mutation present. Additionally, sALS is more complex than that of fALS because the cause of the disease is attributed to an additive effect of hundreds of common variants that increase the risk of developing the disease. Even more, some sALS cases cannot be attributed to genetic or biologic factors instead they are ascribed to environmental and undefined factors (Brown and Al-Chalabi, 2017). Many ALS patients show cognitive and behavioral changes characteristic of frontotemporal dementia (FTD), a neurodegenerative disease that shares neuropathological and genetic features with ALS. Thus, it has been suggested that both FTD and ALS are a continuum of the same phenotypic spectrum (Lipton et al., 2004; Vance et al., 2006; Volk et al., 2018).

Ever since the first causative gene for fALS, Cu/Zn superoxide dismutase 1 (*SOD1*), was discovered in 1993 (Rosen et al., 1993), over 30 genes have been linked to fALS as well as being identified as the molecular cause in certain sALS cases. The list of ALS-related genes is continuously growing, however, *SOD1*, chromosome open reading frame 72 (C9orf72), *TARDBP* (transactive response DNA-binding protein) and *FUS* (fused in sarcoma) are the most well-studied, mainly because they account for the majority of both fALS and sALS cases (Brown and Al-Chalabi, 2017). On the whole, ALS-related genes can be broadly categorized into four groups depending on the cellular pathways in which they are involved: (1) protein homeostasis; (2) RNA homeostasis and trafficking; (3) cytoskeletal dynamics; and (4) mitochondrial function (Mathis et al., 2019). The purpose of this review is to provide insight into several ALS-related genes linked to the disruption of cytoskeletal dynamics. Specially, we will focus on how the disruption of such dynamics potentially triggers axonal degeneration in MNs, impairing their ability to maintain synapses.

## ALS-Related Genes Affecting Cytoskeletal Dynamics

**Figure 1:**
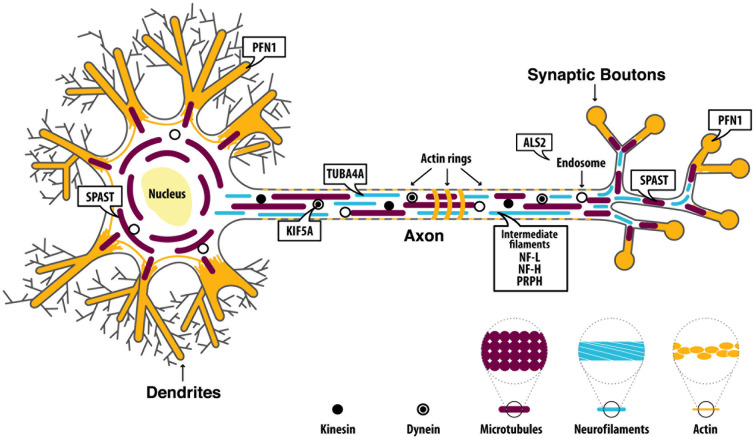
Proteins are encoded by amyotrophic lateral sclerosis (ALS)-related genes affecting cytoskeletal dynamics. ALS2 is mainly implicated in the regulation of endosomal dynamics and it is also found at the growth cone of neurons where it promotes neurite outgrowth. Dynactin Subunit 1 (DCTN1) is involved in microtubules (MTs) anterograde and retrograde transport.Neurofilament light (NF-L), neurofilament heavy (NF-H), andperipherin (PRPH) are the main components of motor neuron (MN) intermediate filaments (IFs). Kinesin family member 5A (KIF5A) encodes a kinesin protein which mainly mediates anterograde transport along MTs. Profilin 1 (PFN1) is involved in the polymerization of actin filaments. Spastin (SPAST) is involved in MTs disassembly and therefore plays a key role in regulating MTs dynamics. Tubulin alpha 4a (TUBA4A) encodes for an α-tubulin subunit which is assembled within the MT filaments.

MNs are known to be the largest polarized cells in the human body and axons of spinal MNs can reach a meter in length in adults. The significant length of such axons makes them highly dependent on proper cytoskeletal architecture, whose integrity is essential for the axonal transport necessary to maintain synapse integrity. Both anterograde (from the cell body to the periphery) and retrograde (from the periphery to the cell body) microtubule (MT)-dependent transport are key mediators for MN survival, maintenance, and functionality (Chevalier-Larsen and Holzbaur, 2006). Thus, disruption of cytoskeleton integrity and/or MT-dependent transport mechanisms could translate into an inability of MNs to supply their synapses with essential components and/or to convey information back to the cell body, potentially triggering degeneration processes. Recently, a few genes known to play a role in cytoskeletal dynamics have been linked to ALS (Figure 1): alsin rho guanine nucleotide exchange factor (*ALS2*), dynactin subunit 1 (*DCTN1*), kinesin family member 5A (*KIF5A*), neurofilament light (*NF-L*), neurofilament heavy (*NF-H*), peripherin (*PRPH*), profilin 1 (*PFN1*), spastin (*SPAST*) and tubulin alpha 4a (*TUBA4A*). Below, we discuss each gene and outline how ALS-associated mutations (Supplementary Table 1) might affect the normal function of each protein, and the implications for the axonal cytoskeletal system.

### Alsin Rho Guanine Nucleotide Exchange Factor (*ALS2*)

*ALS2*, which encodes the protein alsin, is one of the few ALS-related genes that exhibits a recessive pattern of inheritance, often linked to juvenile onset-ALS (Hadano et al., 2001; Yang et al., 2001). Nevertheless, mutations in *ALS2* have also been linked to other diseases selectively affecting MNs, namely infantile ascending hereditary spastic paraplegia (IAHSP) and juvenile primary lateral sclerosis (PLS), which have different clinical profiles to ALS. For instance, in IAHSP and PLS the degeneration is restricted to upper MNs, while both upper and lower MNs are affected in ALS. This broad range of phenotypes arising from mutations in *ALS2* adds complexity to its study (Hand et al., 2003; Simone et al., 2018). Importantly, only two studies report the development of ALS in two independent families as a result of an *ALS2* mutation, making it an extremely rare cause for the development of ALS (Hadano et al., 2001; Yang et al., 2001).

The identified mutations causing an ALS phenotype are deletions producing a premature stop codon, suggesting that alsin loss of function might be triggering disease mechanisms. However, *ALS2* knockout (*ALS2*^−/−^) mice lack an overt pathological phenotype, suggesting that *ALS2* might not contribute to ALS pathology through either a loss of function or a dominant-negative mechanism (Cai et al., 2008). The existence of an alsin-related protein (*ALS2CL*) with homology to the C-terminal domain of alsin found in mice and humans, suggests the possibility of a compensatory mechanism to cope with the loss of function of alsin. However, this hypothesis remains controversial since a few studies imply that the alsin-related protein has a distinct function from alsin (Hadano et al., 2004) and/or it binds to alsin to function as a complex thereby playing a role in other pathways (Suzuki-Utsunomiya et al., 2007). While the loss of alsin appears to be insufficient to trigger MN degeneration (Cai et al., 2005, 2008), its absence can increase MN susceptibility to oxidative stress induced by environmental factors (e.g., paraquat) *in vivo* and *in vitro* (Cai et al., 2005).

Alsin contains three domains that act as GTPase regulators (Hadano et al., 2001): (1) a regulator of chromosome condensation 1 (RCC1) like domain (termed RLD); (2) a diffuse B cell lymphoma (Dbl) homology/pleckstrin homology domain (termed DH/PH); and (3) a vacuolar protein sorting 9 domain (termed VPS9). All three domains of alsin have been implicated in the regulation of endosomal dynamics (Chandran et al., 2007). It has been shown that the genetic ablation of *ALS2* in SOD1^H46R^ mice exacerbates the disruption of endolysosomal trafficking (Hadano et al., 2010), although more studies are needed to analyze the possible interactions between SOD1 and alsin in causing an ALS phenotype. *ALS2* has also been linked to glutamate-mediated excitotoxicity, an ALS synaptic disease mechanism. The RCC1 domain can interact with several domains of the glutamate receptor-interacting protein 1 (*GRIP1*), which mediates GluR2 subunit transport to different cellular compartments. GluR2 is important for AMPA receptors as it makes them calcium impermeable. In *ALS2*^−/−^ neurons, the subcellular localization of GRIP1 protein is altered, which translates into a reduction of GluR2 at the synaptic surface, making MNs more vulnerable to excitotoxicity (Lai et al., 2006). Alsin has also been located at the growth cone of neurons, where it promotes neurite outgrowth (Tudor et al., 2005). Furthermore, when *ALS2* was specifically knocked down in rat embryonic spinal MNs, increased cell death, and reduced neurite outgrowth was observed (Jacquier et al., 2006).

Remarkably, alsin has been found to also have neuroprotective effects in MNs within an ALS context. One study showed that the DH/PH domain of alsin can bind mutated SOD1 and suppress its neurotoxic effects (Kanekura et al., 2004). Currently, no animal models are available to study ALS-related mutations in *ALS2* *in vivo*. Such models would give a broad perspective into the neuroprotective and neurotoxic effects of alsin in ALS.

### Dynactin Subunit 1 (*DCTN1*)

The dynactin complex contains more than 20 subunits, corresponding to 11 different proteins. The largest subunit of dynactin, dynactin subunit 1 or p150^Glued^ (*DCTN1*), directly interacts with cytoplasmic dynein-1 motor (dynein) and MTs to promote the retrograde transport of vesicles, organelles, RNAs, and different binding proteins (Laird et al., 2008; Moughamian and Holzbaur, 2012; Urnavicius et al., 2015). An initial study identified a missense mutation (G59S) in *DCTN1* as the cause of an autosomal dominant form of lower MN disease in all the affected members of one family (Puls et al., 2003). The G59S substitution is localized in the cytoskeleton-associated protein glycine-rich (CAP-Gly) domain of *DCTN1* which mediates the binding of dynactin to MTs. Consistently, subsequent studies showed that G59S mutation decreases the ability of dynactin to bind MTs (Puls et al., 2003; Levy et al., 2006; Lai et al., 2007). Several other mutations in *DCTN1* have now been identified in fALS (Münch et al., 2004, 2005; Liu et al., 2014), and some studies have revealed the presence of *DCTN1* variants in sALS cases. Such variants were able to induce abnormal morphological changes when overexpressed in an *in vitro* system (Stockmann et al., 2013). However, *DCTN1-mRNA* has been observed to be reduced in the motor cortex and spinal cord of sALS patients (Jiang et al., 2007; Ikenaka et al., 2013; Kuźma-Kozakiewicz et al., 2013), raising the question of whether *DCTN1* variants may contribute to disease onset through either a loss or gain of function in ALS.

Transgenic animal models with mutated or ablated *DCTN1* have been generated to elucidate the potential role of *DCTN1* in the degeneration of MNs. The intracellular trafficking of autophagosomes (Laird et al., 2008; Ikenaka et al., 2013) and the bidirectional transport of motor proteins kinesins (Hsu et al., 2011) are disrupted *in vivo*. Remarkably, *DCTN1* has also shown to play a key role within the synaptic processes of MNs. For instance, *DCTN1* depletion leads to neuromuscular junction (NMJ) instability, functional abnormalities, and locomotion defects in a zebrafish model (Bercier et al., 2019). Similarly, the ablation of *DCTN1* in the postnatal neurons of aged mice resulted in the preferential degeneration of spinal MNs, accompanied by increased gliosis, NMJ disintegration, and muscle atrophy (Yu et al., 2018). When *DCTN1* was mutated in Drosophila in the *DCTN1* homolog *Glued*, the formation and maturation of MN synapses were impaired (Allen et al., 1999). Additionally, the dynactin complex was shown to have a local role within the MN presynaptic terminal by controlling synapse stabilization by decreasing the rate of presynaptic retraction (Eaton et al., 2002). Thus, mutations in *DCTN1* can disrupt the local role of dynactin at the MN presynaptic terminal. Taken together, all these studies point towards an inability of MNs to form synapses when *DCTN1* is mutated or ablated. However, the exact mechanisms leading to such deficits remain unknown.

### Intermediate Filament Proteins

Mature MNs express different types of intermediate filament (IF) genes that code for proteins which contribute to the maintenance of the cytoskeletal architecture and signaling within the cells: neurofilament (NF) light (*NF-L*), medium (*NF-M*), and heavy (*NF-H*) chains, as well as α-internexin (*INA*) and peripherin (*PRPH*). These proteins assemble into complex structures and undergo several post-translational modifications that include glycosylation and phosphorylation, particularly NF-H, with the presence of numerous lysine-serine-proline (KSP) repeats in the C-terminal tail domain of the protein that is heavily phosphorylated. Abnormal accumulation of NFs in the spinal cord of patients with sALS was first reported in the 80s (Hirano et al., 1984) and since then, growing evidence has demonstrated that NF abnormalities could be an early pathological feature of ALS in patients, a phenotype that can also be recapitulated in animal models. Perikaryal and axonal inclusion bodies (also termed spheroids) containing IF proteins are a hallmark of degenerating spinal MNs in ALS patients. NF-L and NF-H subunits, as well as peripherin, are particularly enriched in such spheroids, and how they interact has been gaining attention in the last few years.

In 1994, Bergeron and colleagues discovered that *NF-L*-mRNA was decreased by 60% in the spinal MNs of ALS patients (Bergeron et al., 1994). To further study the contribution of *NF-L* in ALS pathogenesis, a transgenic mouse model expressing a low level of human *NF-L* with a point mutation was created [NF-L(Pro)]. These mice exhibited distinct hallmarks of ALS including selective degeneration of MNs, perikaryal and axonal swellings with the presence of IF spheroids, and NMJ denervation presenting as muscle atrophy (Lee et al., 1994). However, there are no known point mutations in *NF-L* associated with ALS, and *NF-L* subunit accumulation is thought to be a secondary effect of other primary disease mechanisms.

In contrast to *NF-L*, mutations in the region of the gene *NF-H* coding for the highly phosphorylated C-terminal domain of the NF-H protein have been reported in several patients with sALS (Figlewicz et al., 1994). Similar to patients, transgenic mice overexpressing human *NF-H* present with features of ALS pathology, including swellings of proximal axons in the spinal cord, progressive axonopathy, and atrophy of muscle fibers (Côté et al., 1993). In these mice, it is not only the axonal transport of NF proteins that are altered but to a lesser extent, also that of actin and tubulin. Also, in degenerating spinal MNs, mitochondria are found within the perikaryon, next to NF aggregates, supporting the idea that disorganization of the NFs architecture can affect the axonal integrity of MNs by altering the transport of other essential components (Collard et al., 1995). These hallmarks of ALS pathology have also been reported in transgenic mice overexpressing various mutations of human *SOD1* and NF involvement in the pathology observed in these models has been previously discussed (Julien, 1997). For instance, transgenic *SOD1*^G93A^ mice that develop an ALS clinical phenotype, present pathological hallmarks of the disease, such as the presence of some NF-rich spheroids containing NF proteins and phosphorylated NF-H and NF-M subunits, as well as α-internexin and peripherin. However, in this case, it seems that only the NF cytoskeleton is altered as no immunoreactivity against actin or tubulin was observed in these spheroids (Tu et al., 1996). To reconcile the role of NF in the *SOD1*-mediated pathology, a transgenic *SOD1*^G37R^ mouse overexpressing human *NF-H* has been generated. Surprisingly, the lifespan of these animals was increased by *NF-H* overexpression (Couillard-Després et al., 1998). In these mice, the NF proteins were primarily localized in the perikaryal area of neurons where NF-H might exert its neuroprotective effect.

While understanding the regulation of IFs can help elucidate the underlying causes of ALS, examining the levels of NF-L and the phosphorylated form of NF-H in cerebrospinal fluid (CSF), plasma, and blood of patients can be used as a diagnostic tool for and as a predictor of disease progression. Indeed, several recent studies with a cohort of ALS patients have reported an increase in these levels in both patients with fALS and sALS. Given their potential as biomarkers for ALS, considerable efforts have recently been made in the development of precise and reliable detection techniques for both NF-L and the phosphorylated form of NF-H (for review, see Poesen and Van Damme, 2018).

Peripherin, encoded by the gene *PRPH*, is a component of MN spheroids in both transgenic mouse models (Beaulieu et al., 2000; Robertson et al., 2003) and ALS patients (Corbo and Hays, 1992; Migheli et al., 1993; Keller et al., 2012). Since ALS patients typically show decreased levels of *NF-L*-mRNA, a transgenic mouse model overexpressing peripherin but with *NF-L* knocked out (TPer;L^−/−^) was created. The TPer;L^−/−^ mouse model exhibited a 46% loss in MNs with spheroids mainly found in the axons and, in the absence of *NF-L*, there was an increase in the formation of *NF-H* heterodimers with peripherin and α-internexin (Beaulieu et al., 1999, 2000). Such spheroids can trigger MN degeneration by disrupting the axonal transport of the other IF proteins as observed in a less complex transgenic mouse model only overexpressing peripherin (Per mouse; Beaulieu et al., 1999; Millecamps et al., 2006). Importantly, the overexpression of *NF-H* within the TPer;L^−/−^ genetic background (hH:TPer;L^−/−^) can protect against the neurotoxicity exerted by peripherin. In these mice, the intracellular inclusion bodies were re-localized to the perikaryal of the spinal MNs, suggesting that excess NF-H sequesters peripherin and by doing so, prevents its accumulation within the axon. This supports the hypothesis that the composition of the IF protein inclusions determines their localization within the cell as well as their role as neuroprotective or neurotoxic structures (Beaulieu and Julien, 2003).

In mice, *PRPH* gives rise to three splice variants (Per 56, 58, 61). The *SOD1*^G37R^ mouse model, in which MNs carry IF spheroids containing peripherin similar to ALS patients, was used to demonstrate that Per61 was the splice variant enriched within the spheroids. However, the existence of the Per61 splice variant in humans remains controversial since it is argued that the splicing event that happens in mice cannot occur in humans (Xiao et al., 2008). Additionally, only one study has been able to find Per61 within lumbar degenerating MNs of two ALS patients (Robertson et al., 2003). Instead, a different splice variant, Per28, is thought to be the equivalent of Per61 in humans and it is upregulated at the mRNA and protein level, in patients with sALS. The analysis of spinal cord sections of these patients showed Per28 aggregates within MNs, and its subsequent overexpression in an *in vitro* system showed Per28 had neurotoxic effects (Xiao et al., 2008). However, the exact role of Per28 in ALS onset remains controversial as it has also been found to have cytoprotective effects against oxidative stress (McLean et al., 2014).

Until 2011, it was thought that peripherin neurotoxicity was a secondary disease mechanism associated with other events like a *SOD1* mutation. However, the identification of several ALS-associated point mutations (Gros-Louis et al., 2004; Leung et al., 2004; Corrado et al., 2011) and a frameshift deletion (Gros-Louis et al., 2004) in *PRPH* raised the question as to whether these mutations are drivers of the ALS phenotype. However, the absence of mouse models or human iPSCs with mutations in *PRPH* has prevented further testing of this hypothesis.

### Kinesin Family Member 5A (*KIF5A*)

Recently, the kinesin family member 5A (*KIF5A*) was confirmed as an ALS-related gene (Nicolas et al., 2018). Kinesins are the microtubule-based motor proteins involved in the anterograde transport of cargos. Currently, it is unknown if *KIF5A* mutations themselves are sufficient to cause ALS but genome-wide analysis has identified *KIF5A* mutations as low or high-risk factors for the development of ALS (Nicolas et al., 2018). The mechanisms through which *KIF5A* mutations would be contributing to ALS onset have not been studied yet, but several hypotheses exist focused on the central role of kinesins in axonal transport. For instance, *KIF5A* knockout mice (*KIF5A*^−/−^) display abnormal transport of NF proteins (Xia et al., 2003), which has been proposed as a causative mechanism of NF accumulation, an ALS hallmark, as discussed previously (Chevalier-Larsen and Holzbaur, 2006). Additionally, primary motor neurons (PMNs) derived from *KIF5A*^−/−^ mice showed transport deficits, reduced axonal outgrowth, and reduced survival. In particular, such transport deficits were observed for mitochondria (Karle et al., 2012). The impairment of mitochondrial transport was observed in both anterograde and retrograde direction, consistent with previous findings in *Drosophila* models lacking the *KIF5A* homolog *khc* (Martin et al., 1999). Importantly, deficits in mitochondria transport and function have also been identified as hallmarks of ALS (Chevalier-Larsen and Holzbaur, 2006; Smith et al., 2019). *KIF5A* also has been shown to affect neurite outgrowth through its interaction with protrudin in the mouse brain, which plays a role in the regulation of vesicular transport in neurons (Matsuzaki et al., 2011). Impaired neurite outgrowth could potentially affect the ability of MNs to form synaptic connections, which are lost in ALS.

### Profilin 1 (*PFN1*)

Profilin 1 (*PFN1*) is an essential protein for the polymerization of filamentous (F)-actin through binding of monomeric (G)-actin. Early studies assessing the impact of *PFN1* mutations in different neuronal cell types showed that a profilin mutant (H119E), which conserved its ability to bind all its target proteins except actin, blocked neurite formation *in vitro* (Suetsugu et al., 1998). Later, *in vivo* studies in *Drosophila* showed growth cone arrest and reduced axon outgrowth in embryonic MNs carrying a mutation in *chickadee*, the homolog to *PFN1* (Wills et al., 1999). In 2012, the exome sequencing of two large ALS families displaying a dominant pattern of inheritance and the subsequent screening of a larger cohort revealed a link between ALS and several mutations in *PFN1* (C71G, M114T, E117G, G118V). In the same study and consistent with early findings, PMNs overexpressing mutated *PFN1* (G118V) demonstrated a reduction in levels of bound actin relative to wild-type *PFN1*, inhibition of axonal outgrowth, and growth cone size reduction (Wu et al., 2012). Since then, additional ALS cohorts have been analyzed and further mutations in the *PFN1* gene have been identified, not only in fALS but also in sALS (Ingre et al., 2013; Tiloca et al., 2013; Yang et al., 2013; Smith et al., 2015).

Aside from dysregulation of actin dynamics that disrupt axonal growth and promote growth cone arrest, *PFN1* has been linked to other ALS features that include abnormalities in autophagy (Nguyen et al., 2019) and cytoplasmatic aggregations (Wu et al., 2012; Smith et al., 2015). *PFN1* aggregates co-stained for the transactive response DNA-binding protein 43 (TDP-43) *in vitro*. Interestingly, abnormal PFN1 pathology was not observed in a cohort of sALS patients with TDP-43 pathology, suggesting that whereas mutant PFN1 can induce aggregation of TDP-43, PFN1 aggregation does not occur in patients with TDP-43 pathology. The mechanisms inducing TDP-43 accumulation through mutant PFN1 remains unknown (Wu et al., 2012). TDP-43 is encoded by *TARDBP*, another ALS-related gene ubiquitously expressed in the majority of cells, which plays a key role in the regulation of RNA metabolism in different subcellular compartments (Brown and Al-Chalabi, 2017; Mathis et al., 2019). Further studies are needed to analyze the possible interaction between these two genes in disease onset in sALS.

Transgenic mice models of *PFN1* have been generated to study the contribution of this gene to ALS pathology. For instance, mice harboring the *PFN1* G118V variant display several clinical and pathological characteristics of ALS, including loss of lower and upper MNs, loss of NMJs, and profilin aggregation. Consistent with profilin function and results obtained in PMNs overexpressing the same mutation in *PFN1*, these mice also show an abnormal G/F actin ratio in the spinal cord (Fil et al., 2017). Similarly, another mouse model in which mutated *PFN1* (C71G) was restricted to MNs during development showed abnormal G/F actin ratio in the spinal cord and significant motor deficits (Brettle et al., 2019).

### Spastin (*SPAST*)

Spastin protein, coded by the gene *SPAST* (or *SPG4*) is a member of the ATPases associated with diverse cellular activities (AAA) family that can induce MT severing *in vitro*, thereby influencing MT dynamics (Errico et al., 2002). In support of this observation, overexpression of spastin increases MT disassembly, negatively affecting axonal transport (Kasher et al., 2009). Over 150 mutations within the *SPAST* gene have been identified to date, most of them being causative of hereditary spastic paraplegia (HSP) but two mutations have been associated with an ALS phenotype (Meyer et al., 2005; Münch et al., 2008). The first ALS reported case linked to the SPG4 gene was the result of a duplication mutation within exon 1 (Meyer et al., 2005). Unlike the duplication mutation that gave rise to an early-onset but slowly progressing ALS, a missense mutation *in* *SPAST* (S44L) gave rise to a rapidly progressive adult-onset ALS (Münch et al., 2008). Excitability studies were performed to identify the cortical excitability changes in HSP, ALS, and PLS patients. The three diseases were shown to have different patterns of cortical excitability, ALS is characterized by cortical hyperexcitability. Nevertheless, the molecular mechanisms behind the different excitability found in HSP and ALS remain unknown (Geevasinga et al., 2015). So far, there are no studies of spastin in ALS. However, *SPAST* mutations leading to an HSP phenotype display a dying-back axonopathy of the affected neurons, especially the corticospinal MNs. Moreover, such axon abnormalities have been identified in iPSC-derived neurons (Denton et al., 2014, 2016), which are known to be a powerful tool to study human neurons *in vitro*.

### Tubulin Alpha 4a (*TUBA4A*)

Genetic screening of several fALS (Smith et al., 2014; Li et al., 2018) and sALS (Pensato et al., 2015) patients has revealed several mutations in the *TUBA4A* gene, encoding the tubulin alpha 4a protein, a ubiquitously expressed MT protein highly enriched in the nervous system but lacking a known role in MNs (Rustici et al., 2013; Smith et al., 2014). Through several *in vitro* experiments, Smith et al. (2014) demonstrated that most of the identified variants showed the inefficient formation of α-/β- tubulin dimers, decreased incorporation into MTs, and inhibited MT network stability. However, it remains controversial whether the proteins resulting from *TUBA4A* mutations can form aggregates since only one familial ALS mutation (W407X) has been shown to trigger the formation of small ubiquitinated cytoplasmic inclusions *in vitro* in PMNs and HEK293T cells (Smith et al., 2014).

Unlike other MT proteins linked to neurological disorders, *TUBA4A* expression increases dramatically (>50-fold) with age in humans, potentially explaining why a mutation in this gene may promote a late-onset disease (Tischfield et al., 2011; Smith et al., 2014; Clark et al., 2016). Interestingly, decreased levels of *TUBA4A*-mRNA have been found in the brain and spinal cord of sALS and fALS patients with mutations in *SOD1* and *C9orf72* (Helferich et al., 2018). Unfortunately, the impact of *TUBA4A* mutations in MT dynamics and how its expression changes over time have not been assessed *in vivo*. Recently, a neuron-like cell line with transient overexpression of ALS-related mutated forms (R320C and A383T) of *TUBA4A* showed altered neurite length and MT defects after exposure to selenium (Maraldi et al., 2019). This novel study highlighted a potential link between environmental factors and TUBA4A mutations in triggering ALS onset. The development of animal models harboring ALS mutations in the *TUBA4A* gene will help lead to understanding the role of *TUBA4A* in ALS.

## Targeting Cytoskeletal Dynamics to Treatment for ALS

Currently, neither the FDA-approved drugs for ALS nor the drugs at different stages of clinical trials are known to have a direct effect on the dysfunction of cytoskeletal dynamics. Besides the IF proteins (NF-L and NF-H subunits, and peripherin) which aggregate to form perikaryal or axonal spheroids (Hirano et al., 1984; Corbo and Hays, 1992; Côté et al., 1993), no other connection between any of the aforementioned proteins has been described. For many years, the dysfunction of most of these proteins was thought to be a result of mutations in other genes (e.g., *SOD1*) that trigger the disruption of several cellular processes within an MN (Julien, 1997; Couillard-Després et al., 1998; Xiao et al., 2008; Hadano et al., 2010). However, this perspective is now challenged. The discovery of mutations able to trigger disease mechanisms, in the different genes affecting cytoskeletal dynamics, has increased the consensus on a greater contribution of such genes in the development of ALS. Therefore, in addition to NF-L and the phosphorylated form of NF-H that have recently been identified as disease markers (Poesen and Van Damme, 2018), these genes and their products have the potential to be studied as clinical targets.

## Conclusion

The discovery of ALS-related genes that affect cytoskeletal dynamics is very recent and their study in an ALS-related context does not extend more than 20 years. The available studies are mainly descriptive reports of isolated subjects or one family with a few members. Only a couple of genetic studies count with large cohorts of patients and/or an extensive study model (e.g., GWAS), meaning that the population presence of most of the mutations remains unknown. It is still unclear which of these genes are acting as the primary cause in the onset of ALS. Some studies identified mutations in genes only in patients with sALS or fALS and not in controls. In contrast, others identified some mutations in patients as well as in non-affected individuals, while others have failed to report any significant disease-associated mutations in the same genes. For example, variants in NF genes are mainly considered a risk factor but a larger cohort of patients might have to be considered to conclude if specific variants are the primary cause of ALS or simply a risk factor. Instead of triggering disease onset or acting as risk factors, different mutations in ALS-related genes can also act as modifiers of the disease by changing the age of clinical presentation (early VS late-onset), the evolution of the disease (e.g., fast VS slow progression), the development of certain profiles (e.g., mutations on *ALS2* or mutations on *DCTN1* observed within the ALS/FTD family case), the molecular changes observed, etc. Additionally, the interaction between two or more of these genes to trigger and/or modify the clinical presentation of the disease has to be taken into account. It is also important to mention that not all the studies systematically looked at several ALS-related genes in the same study, meaning that the possibility exists that a mutation might be associated as the cause of the disease while not accounting for the presence of other mutations in other genes that might be playing a more significant role in the development of ALS. As to the molecular mechanisms through which the different variants of these genes might be contributing to ALS onset and progression, most of them remain poorly understood. Therefore, animal models harboring ALS-related mutations have been generated to study disease mechanisms, however, they exist for only a few of these genes. Similarly, there are almost no studies making use of novel approaches such as iPSC technology and CRISPR-Cas9 to study the impact of these mutations in ALS pathology. Due to their large size, MNs highly rely on cytoskeletal dynamics to maintain axonal transport and synapse integrity which allows them to function properly. Efforts to study how the ALS-related genes are linked to abnormalities of cytoskeletal dynamics should be increased to better understand the mechanisms underlying this lethal disease.

## Author Contributions

MC-M contributed with the conception and design of the manuscript, as well as in the drafting and substantial revision. MC contributed to the drafting and substantial revision of the manuscript. TD contributed with substantial revision of the manuscript. All authors contributed to the article and approved the submitted version.

## Conflict of Interest

The authors declare that the research was conducted in the absence of any commercial or financial relationships that could be construed as a potential conflict of interest.
