# Supplementary material for: The Neglected Genes of ALS: Cytoskeletal Dynamics Impact Synaptic Degeneration in ALS
Source: Front Cell Neurosci. 2020 Nov 13;14:594975. doi: 10.3389/fncel.2020.594975 (PMC7691654; doi:10.3389/fncel.2020.594975)
Supplement: Supplementary file 1 [file Table_1.docx]

**Supplementary Table 1.** Summary of mutations on ALS-related genes

| **Gene** | **Variant** | **Method of detection** | **Study model** | **fALS/sALS** | **Degree of pathogenicity** | **Clinical profile** | **Presence of inclusions** | **Relevant experimental data*** | **Reference**  **(description of the mutation)** |
| --- | --- | --- | --- | --- | --- | --- | --- | --- | --- |
| *ALS2* | 261delA  Produces loss of function | Sanger sequencing | Haplotype analysis | fALS | n/a | Early onset (between 3 and 10 years old)  Increased loss of upper MN and progressive spasticity of limbs, facial and pharyngeal muscles | unknown | *ALS2*^-/-^ mice lack an overt phenotype  Absence of alsin in *SOD1-*H46R mice disrupts endolysosomal trafficking  *ALS2* knockdown increased cell death and reduced neurite outgrowth in rats  *In vitro*, the absence of alsin increases susceptibility to oxidative stress in *ALS2*^-/-^ neuron  *In vitro*, the absence of alsin increases vulnerability to excitotoxicity in *ALS2*^-/-^ neurons | Hadano *et al.,* 2001 |
|  | 1548delAG  Produces loss of function |  |  |  | n/a | Early onset (between 3 and 10 years old)  Similar to 261delA clinical profile but without evidence of early denervation | unknown |  |  |
| *DCTN1* | G59S | Sanger sequencing | GWLA | fALS | High penetrance | Early onset (35 years old)  Slowly progressive lower MN disease with bulbar onset and without sensory symptoms | unknown | *In vivo* and *in vitro DCTN1*-G59S decreases the ability of dynactin to bind MTs  *In vitro,* MN1 cells transfected with GFP-tagged G59S p150Glued show increased cell death and aggregations of the mutant protein in the cell body (associated with mitochondria). The aggregations co-stained with DIC, GM130 and the 20S proteasome. Similar effects are observed in pMNs transfected with the same construct where evidence of ubiquitination can also be found | Puls *et al*., 2003 |
|  | M571T | Heteroduplex analysis and dsDNA sequencing | n/a | fALS | n/a | Early onset (48 years old)  Spinal onset | unknown | Transfection of pMNs and COS7 cells with the M571T-GFP-p150 construct did not produce major changes in cellular morphology. | Münch *et al.,* 2004 |
|  | R785W | Heteroduplex analysis and dsDNA sequencing | n/a | fALS  (also present in controls) | Incomplete penetrance | Early onset (between 55 and 64 years old)  Spinal or bulbar onset | unknown | Transfection of pMNs and COS7 cells with the R785W-GFP-p150 construct did not produce major changes in cellular morphology. | Münch *et al.,* 2004 |
|  | R1101K | Heteroduplex analysis and dsDNA sequencing | n/a | fALS | n/a | Early onset (between 55 and 61 years old).  ALS/FTD family (some members presented with MN disease symptoms while others only presented dementia symptoms) | unknown | Transfection of pMNs and COS7 cells with the R1101K-GFP-p150 construct did not produce major changes in cellular morphology | Münch *et al.,* 2005 |
|  | D63Y | Haloplex exome target enrichment system for Illumina sequencing | n/a | sALS  (also present in controls) | n/a | n/a | unknown | Transfection of pMNs with the D63Y-GFP-p150 construct promotes formation of p150 aggregates in the cytoplasm and neurites that become larger with time. Evidence of ubiquitination is present | Stockmann *et al.,* 2013 |
|  | E34Q |  |  |  | Incomplete penetrance |  | unknown | Transfection of pMNs with the E34Q-GFP-p150 promotes formation of p150 aggregates (to a lesser extent than D63Y) in the cytoplasm and neurites that become larger with time |  |
| *PFN1* | C71G | Exome sequencing | Linkage analysis | fALS | High penetrance | Early onset (between 40 and 50 years old)  Spinal onset | unknown | Cytoplasmic aggregates of the mutant PFN1 protein in N2A cells and pMNs co-stained with TDP-43  Mutant protein detected in the insoluble fraction with increased insoluble mutant PFN1 protein levels following proteasome inhibition  Decreased axon outgrowth, reduced growth cone size | Wu *et al.,* 2012 |
|  | M114T |  |  |  |  | Early onset (between 42 and 52 years old)  Spinal onset | unknown | Cytoplasmic aggregates of the mutant PFN1 protein in N2A cells and pMNS  Mutant protein detected in the insoluble fraction with increased insoluble mutant PFN1 protein levels following proteasome inhibition  Decreased axon outgrowth |  |
|  | G118V |  |  |  |  | Early onset (43 years old)  Spinal onset | unknown | Cytoplasmic aggregates of the mutant PFN1 protein in N2A cells and pMNs co-stained with TDP-43  Mutant protein detected in the insoluble fraction with increased insoluble mutant PFN1 protein levels following proteasome inhibition  Decreased axon outgrowth, reduced growth cone size |  |
|  | E117G | Exome sequencing  Sanger sequencing  Sanger sequencing  Sanger sequencing | Linkage analysis  Case-control meta-analysis of all reported E117G cases  n/a  n/a | fALS and sALS  fALS and sALS  sALS  fALS | n/a  Low penetrance  n/a  n/a | Early onset (40 years old for fALS, between 34 and 63 years old for sALS)  Between 54 and 74 years old  Spinal onset  Late onset (73 years old)  Bulbar onset  Early onset (54 years old)  Spinal onset | unknown  Neuronal and or/ glial p62 positive, TDP-43 positive cytoplasmic inclusions  unknown  unknown | Cytoplasmic aggregates of the mutant protein in N2A cells but not significant  Moderate increased insoluble mutant PFN1 protein levels following proteasome inhibition  Diffuse cytoplasmic staining of mutant PFN1 in transfected HEK293T    No increase in the insolubility of mutant PFN1 in HEK293T cells | Wu *et al.,* 2012  Smith *et al.*, 2015  Tiloca *et al.*, 2013  Ingre *et al*., 2013 |
|  | A20T | Sanger sequencing | n/a | fALS | n/a | 63 years old  Spinal onset  Predominant lower MN phenotype | unknown | Increased percentage of cells with granular staining for mutant PFN1 in transfected HEK293T  Increased insolubility of mutant PFN1 in HEK293T cells  Increased insoluble protein levels of mutant PFN1 following proteasome inhibition in patient’s fibroblasts | Smith *et al.*, 2015 |
|  | Q139L |  |  | sALS |  | Early onset (52 years old) | Glial p62 positive, TDP-43 positive cytoplasmic inclusions in the cord and motor cortex | Diffuse cytoplasmic staining of mutant PFN1 in transfected HEK293T  No increase in the insolubility of mutant PFN1 in HEK293T cells |  |
|  | R136W | Sanger sequencing | n/a | sALS | n/a | 27 years old (limb onset) | unknown | None | Chen *et al.,* 2013 |
|  | T109M | Sanger sequencing | n/a | fALS | Incomplete penetrance | Early onset (48 years old)  Spinal onset | unknown | None | Ingre *et al*., 2013 |
| IFs |  | | | | | | | | |
| *PRPH* | 228delC | Sanger sequencing | n/a | sALS | n/a | 60 years old  Spinal onset | unknown | IF assembly disruption by the expression of the mutant protein in SW13 cells | Gros-Louis *et al*., 2004 |
|  | D141Y | Sanger sequencing  Sanger sequencing  Heteroduplex analysis and dsDNA sequencing | n/a  n/a  n/a | sALS  sALS (also observed in controls)  n/a | n/a  n/a  Low penetrance | Early onset (42 years old)  Spinal onset  Early onset (56 years old)  n/a | Moderate loss of MNs and astrocytosis in the spinal cord  Large filamentous  inclusions containing peripherin and  NF-L  unknown  unknown | Presence of filamentous aggregates in SW13 cells transfected with D141Y mutant that also contains NF-L when both proteins are co-expressed | Leung *et al*., 2004  Gros-Louis *et al*., 2004  Corrado *et al*., 2011 |
|  | R133P | Heteroduplex analysis and dsDNA sequencing | n/a | sALS | n/a | Late onset (70 years old)  Spinal onset | unknown | Predicted to have deleterious effect on the protein structure/function (bioinformatics analysis) | Corrado *et al*., 2011 |
| *NF-H* | **deletions**  **in the tail domain**  Δ790  Δ528-561 | Amplification and sequencing of the C-terminal region (KSP repeats) | n/a | sALS | n/a | Between 46 and 65 years old  Late onset 72 years old | unknown | None | Figlewicz *et al*., 1994 |
|  | Δ663-668  Δ655-662  Δ663-677  Δ743-748 | Amplification and sequencing of the C-terminal region (KSP repeats) | n/a | sALS  sALS  fALS (not established, also observed in unaffected members of the family)    fALS (not established, also observed in unaffected members of the family) | n/a | Late onset (73 years old  Spinal onset  Late onset (66 years old)  Spinal onset  Late onset (66 years old)  Spinal onset  Early onset (50 years old)  Spinal onset | unknown | None | Al-Chalabi *et al.,* 1999 |
|  | **insertion in the tail domain 28aa**  ins714 | Amplification and sequencing of the C-terminal region (KSP repeats) | n/a | sALS (not established) | n/a | Late onset (68 years old)  Spinal onset | unknown | None | Tomkins *et al.,* 1998 |
|  | A380T | Systematic DNA sequence analysis of all three NF genes | n/a | sALS | n/a | n/a | unknown | None | Garcia *et al.,* 2006 |
| *SPAST* | S44L | single strand conformation analysis  (SSCP) and direct DNA sequencing | n/a | sALS | n/a | Early onset (48 years old)  Spinal onset | unknown | None | Münch *et al*., 2008 |
|  | in frame 102insAS | single strand conformation analysis  (SSCP) and direct DNA sequencing | n/a | sALS | n/a | Early onset (24 years old)  Slowly progressive upper and lower MN  syndrome) | unknown | None | Meyer *et al*., 2005 |
|  | N542G | Sanger sequencing | n/a | sALS | n/a | Early onset (33 years old)  Spinal onset | unknown | None | Brugman *et al.* 2005 |
| *KIF5A* | P986L  Produces loss of function | Exome sequencing | GWAS | n/a | Low penetrance | n/a | unknown | *In vivo, KIF5A-/-* mice show abnormal transport of NF proteins  *In vitro,* primary neurons from *KIF5A-/-* display transport deficits for mitochondria, reduced axonal outgrowth and reduced survival | Nicolas *et al.* 2018 |
|  | 2996delA  Produces loss of function |  |  | fALS | High penetrance | Early onset (45 years old)  Slowly progressive | unknown |  |  |
|  | 2993-3C>T  Produces loss of function |  |  | fALS | High penetrance | Early onset (29 years old)  Limb onset  Slowly progressive | unknown |  |  |
|  | 2996delA  Produces loss of function |  |  | fALS | High penetrance | Early onset (42 years old)  Spinal onset | unknown |  |  |
|  | 3019A>G  Produces loss of function |  |  | fALS | High penetrance | Early onset (53 years old)  Spinal onset | unknown |  |  |
|  | 3020G>A  Produces loss of function |  |  | fALS | High penetrance | Early onset (50 years old)  Spinal onset  Slowly progressive | unknown |  |  |
|  | 3020+1G>A  Produces loss of function |  |  | fALS | High penetrance | Early onset (45 years old)  Bulbar onset  Slowly progressive | unknown |  |  |
|  | 3020+2T>A  Produces loss of function |  |  | fALS | High penetrance | Early onset (46 years old)  Bulbar onset  Slowly progressive | unknown |  |  |
|  | 3020+3A>G  Produces loss of function |  |  | fALS | High penetrance | Early onset (50 years old)  Bulbar onset | unknown |  |  |
|  | 291+5A>G  Produces loss of function |  |  | sALS | High penetrance | n/a | unknown |  |  |
|  | 2989delA  Produces loss of function |  |  | sALS | High penetrance | Early onset (50 years old)  Spinal onset  Slowly progressive | unknown |  |  |
|  | 2993-1G>A  Produces loss of function |  |  | sALS | High penetrance | Early onset (52 years old)  Bulbar onset | unknown |  |  |
| *TUBA4A* | R320C | Exome sequencing | Exome-wide rare variant analysis | fALS | n/a | 64 years old  Spinal onset | unknown | *In vitro,* TUBA4A-R320C ability to form tubulin dimers is decreased  *In vitro,* transient overexpression of *TUBA4A-R320C* altered neurite length and MT defects after exposure to selenium | Smith *et al.* 2014 |
|  | R320H |  |  |  |  | Early onset (41 years old)  Spinal onset | unknown | *In vitro,* TUBA4A-R320H ability to form tubulin dimers is decreased  *In vitro,* TUBA4A-R320H has a different distribution within the MT network related to the wild-type |  |
|  | W407X |  |  |  |  | Late onset (66 years old)  Spinal onset | unknown | *In vitro,* pMNs and HEK293T cells transfected with hemagglutinin (HA)-tagged *TUBA4A* W407X construct show aggregate-like inclusions  *In vitro*, TUBA4A-W407X ability to form tubulin dimers is decreased  *In vitro,* TUBA4A-W407X show decreased incorporation into MTs.  *In vitro,* transient overexpression of *TUBA4A-W407X* altered neurite length and MT defects after exposure to selenium |  |
|  | A383T |  |  |  |  | Late onset (71 years old)  Spinal onset | unknown | *In vitro,* TUBA4A-A383T has a different distribution within the MT network related to the wild-type. |  |
|  | R215C |  |  |  |  | Late onset (78 years old)  Spinal onset    FTD/ALS | unknown | *In vitro, TUBA4A* R215C ability to form tubulin dimers remains unchanged. |  |
|  | V7I | Exome sequencing | Exome-wide case-control burden analysis | sALS | n/a | n/a | unknown | None | Pensato *et al*. 2015 |
|  | T349S |  |  |  |  | 62 years old  Bulbar onset | unknown |  |  |
|  | D438N |  |  |  |  | 59 years old  Bulbar onset | unknown |  |  |
|  | 226+4A>G |  |  |  |  | Early onset (14 years old)  Spinal onset  Mild cognitive impairment | unknown |  |  |

*Relevant experimental data section contains the experimental observations of several studies along with the one where the mutation was described for the first time.

When the information was not applicable or not available, n/a was written.

**Supplementary references** (includes references that cannot be find on the main text)

Al-Chalabi, A., Andersen, P. M., Nilsson, P., Chioza, B., Andersson, J. L., Russ, C., Shaw, C. E., Powell, J. F. and Leigh, P. N. (1999). Deletions of the heavy neurofilament subunit tail in amyotrophic lateral sclerosis. *Hum Mol Genet* **8,** 157-164.

Brugman, F., Wokke, J. H., Scheffer, H., Versteeg, M. H., Sistermans, E. A. and van den Berg, L. H. (2005). Spastin mutations in sporadic adult-onset upper motor neuron syndromes. *Ann Neurol* **58,** 865-869.

Chen, Y., Zheng, Z. Z., Huang, R., Chen, K., Song, W., Zhao, B., Chen, X., Yang, Y., Yuan, L. and Shang, H. F. (2013). PFN1 mutations are rare in Han Chinese populations with amyotrophic lateral sclerosis. *Neurobiol Aging* **34,** 1922.e1921-1925.

Garcia, M. L., Singleton, A. B., Hernandez, D., Ward, C. M., Evey, C., Sapp, P. A., Hardy, J., Brown, R. H., Jr. and Cleveland, D. W. (2006). Mutations in neurofilament genes are not a significant primary cause of non-SOD1-mediated amyotrophic lateral sclerosis. *Neurobiol Dis* **21,** 102-109.

Tomkins, J., Usher, P., Slade, J. Y., Ince, P. G., Curtis, A., Bushby, K. and Shaw, P. J. (1998). Novel insertion in the KSP region of the neurofilament heavy gene in amyotrophic lateral sclerosis (ALS). *Neuroreport* **9,** 3967-3970.
